# Near-Infrared Autofluorescence for Parathyroid Detection During Endocrine Neck Surgery: A Randomized Clinical Trial

**DOI:** 10.1001/jamasurg.2025.2233

**Published:** 2025-07-16

**Authors:** Alexandria G. Cousart, Colleen M. Kiernan, Parker A. Willmon, Giju Thomas, Tracy S. Wang, Paul G. Gauger, Quan-Yang Duh, Hunter J. Underwood, Anee Jackson, Anuradha Patel, Anita Mahadevan-Jansen, Carmen C. Solórzano

**Affiliations:** 1 Vanderbilt Biophotonics Center, Vanderbilt University, Nashville, Tennessee; 2 Department of Biomedical Engineering, Vanderbilt University, Nashville, Tennessee; 3 Department of Surgery, Vanderbilt University Medical Center, Nashville, Tennessee; 4 Department of Surgery, Medical College of Wisconsin, Milwaukee; 5 Department of Surgery, University of Michigan, Ann Arbor; 6 Department of Surgery, University of California, San Francisco

## Abstract

**Importance:**

Inadvertent removal and damage to parathyroid glands (PGs) can lead to hypoparathyroidism, making it crucial to accurately identify and preserve these glands during parathyroidectomy and thyroidectomy.

**Objective:**

To assess if fiber-based near-infrared autofluorescence (NIRAF) increases the number of intraoperatively identified PGs and reduces the occurrence of hypoparathyroidism.

**Design, Setting, and Participants:**

This multicenter randomized clinical trial with a 6-month follow-up was conducted between March 2020 and July 2024. It included 4 medical centers across the US, 4 senior (more than 10 years of experience) and 3 junior (less than 5 years of experience) surgeons. A total of 754 patients were enrolled and 752 were randomized, including 398 patients (2 withdrew) who underwent parathyroidectomy and 354 patients who had total/completion thyroidectomy. Data were analyzed from March 2020 to January 2025.

**Interventions:**

Use of fiber-based NIRAF during thyroidectomy and parathyroidectomy.

**Main Outcomes and Measures:**

The primary outcome was the mean number of PGs identified intraoperatively. The secondary outcome was the rate of hypoparathyroidism (transient and at last follow-up).

**Results:**

Of 752 patients randomized, 712 were analyzed for the primary outcome (94.4%) (overall median [IQR] age, 59 [25] years; 516 females [68.4%]). A total of 161 underwent parathyroidectomy with NIRAF, while 159 had conventional surgery. Additionally, 176 underwent thyroidectomy using NIRAF and 178 had traditional surgery. The mean number of PGs identified during parathyroidectomy was not significantly higher when using NIRAF for focused procedures (mean, NIRAF, 1.6; 95% CI, 1.4-1.8 vs control, 1.5; 95% CI, 1.4-1.7). During bilateral explorations, the surgeons improved in the mean number of PGs identified when using NIRAF (mean NIRAF, 3.5; 95% CI, 3.4-3.7 vs control, 3.2; 95% CI, 3.0-3.4; *P* < .001). During thyroidectomy, the mean number of PGs identified increased when using NIRAF (mean NIRAF, 3.3; 95% CI, 3.2-3.4 vs control, 2.8; 95% CI, 2.7-3.0; *P* < .001). There was no significant difference in hypoparathyroidism after thyroidectomy, either transient (NIRAF: 48 of 173 patients [27.8%]; control: 44 of 169 patients [26%]) or at the last follow-up (NIRAF: 3 of 176 patients [1.7%]; control: 6 of 176 patients [3.4%]).

**Conclusions and Relevance:**

Fiber-based NIRAF can increase the number of PGs identified during thyroidectomy and bilateral exploration parathyroidectomy without increasing the duration of the surgery.

**Trial Registration:**

ClinicalTrials.gov Identifiers: NCT05579782, NCT05022667, NCT05022641, NCT04281875, NCT04299425, NCT05152927

## Key Points

**Question:**

Can fiber-based near-infrared autofluorescence (NIRAF) help identify more parathyroid glands (PGs) intraoperatively than standard surgical protocol?

**Findings:**

In this randomized trial of 754 adults, NIRAF during parathyroidectomy was associated with a higher average number of PGs identified per patient when a bilateral exploration was performed (3.2 control vs 3.5 NIRAF per patient). During thyroidectomy, surgeons, on average, identified 17.9% more PGs (3.3 per patient) when using NIRAF than the control (2.8 per patient).

**Meaning:**

Fiber-based NIRAF can increase the number of PGs identified per patient during endocrine neck surgery and may limit the risk of adverse outcomes.

## Teaser

This study assesses if fiber-based near-infrared autofluorescence increases the number of intraoperatively identified parathyroid glands and reduces the occurrence of hypoparathyroidism.

## Introduction

Postsurgical hypoparathyroidism can detrimentally impact quality of life as symptoms can vary from neuromuscular irritability to cardiac arrhythmias and kidney insufficiency.^1,2,3,4^ Risk factors for hypoparathyroidism include inadvertent parathyroid gland (PG) excision, PG autotransplant, and low-postoperative parathyroid hormone (PTH) levels.^5^ The incidence of transient hypoparathyroidism ranges from 9.1% to 32.1% in in patients who undergo thyroidectomy, while permanent hypoparathyroidism affects 0% to 4.2%.^6,7,8,9,10^ During parathyroidectomy, damaging healthy PGs can disrupt calcium homeostasis, resulting in hypocalcemia.^11^ Thus, it is crucial to identify PGs during both procedures to prevent hypoparathyroidism.^1^

The standard for preserving PGs is visual inspection, but this subjective method has severe limitations due to the glands’ small size, variable location, and anatomical variations.^12,13,14,15^ Histopathological confirmation, such as frozen sections (FS) or fine-needle aspirations, are commonly used to verify PGs.^16,17^ However, these methods can add additional time and costs to the surgical procedure.^18^ Near-infrared autofluorescence (NIRAF) has gained attention for its real-time label-free approach for intraoperative PG identification.^19,20,21,22,23,24^

PGs emit a distinct NIRAF signal,^20,21,25,26^ as shown in US Food and Drug Association-granted image-based and fiber-based systems higher than thyroid and other neck tissues.^4,14,16,17,18,19^ Using a fiber-based platform, NIRAF can objectively quantify PG autofluorescence with more than 90% sensitivity and specificity.^21,27,28^ Randomized clinical trials with image-based NIRAF correlate with lower inadvertent parathyroidectomy rates,^8,29^ more PGs identified,^8,30,31,32^ and lower autotransplant rates.^30,31^ This article reports the first multicenter randomized clinical trial of fiber-based NIRAF to determine if quantitative NIRAF improves PG identification (primary outcome), decreases hypoparathyroidism, reduces operative time and FS use, and minimizes inadvertent PG removal (secondary outcomes).

## Methods

### Trial Design

This randomized clinical trial was conducted at 4 medical centers in the US between March 2020 and July 2024. All sites adhered to a shared study protocol coordinated by Vanderbilt University. Eligible patients were adults (18 years or older) undergoing initial or reoperative surgery for primary hyperparathyroidism (parathyroidectomy) or thyroid disease (total/completion thyroidectomy). Patients with secondary/tertiary hyperparathyroidism, pregnancy, partial thyroidectomy, concurrent parathyroidectomy/thyroidectomy, or incidental parathyroid disease were excluded. Patients were randomized 1:1 to receive NIRAF guidance or standard surgical care. Surgical procedures were performed by 4 senior surgeons (more than 10 years of experience) and 3 junior surgeons (less than 5 years of experience). The study was approved by the institutional review boards of all participating centers and written informed consent was obtained from all participants before enrollment. The study followed Consolidated Standards of Reporting Trials (CONSORT) reporting guidelines. This trial was registered at ClinicalTrials.gov (NCT05579782, NCT05022667, NCT05022641, NCT04281875, NCT04299425, NCT05152927).

### Randomization and Masking

Patients were randomly assigned to NIRAF or control groups by block randomization (20 patients per block) using Random Allocation Software.^33^ The study coordinator (P.W.) generated and gave each center a random allocation sequence. Before the procedure, the surgeon consented patients and assigned them to study arm. The patients were blinded to the study assignment.

### Procedures

Surgeons followed standard procedures until a suspected PG was found, then rated their confidence (high, medium, low) that the tissue was PG and completed the surgery in the control group. Surgeons used fiber-based NIRAF, the PTeye (Medtronic), to aid in confirming visually suspected PGs. Baseline autofluorescence was calibrated using thyroid or surrounding tissues; each subsequent measurement was normalized to the baseline as indicated by the detection ratio. A detection ratio of 1.2 or more indicates possible PG and surgeons rerated their confidence in PG identification after device use. Trainees, when present at 3 centers, were asked to rate their confidence similarly. The trial protocol is available in the eMethods in Supplement 1.

### Outcomes

The study’s primary objective is to compare the average number of PGs identified with high confidence per patient with and without NIRAF. As outlined in the eTable in Supplement 1, a normalization method was used to account for instances where PGs were missing.

**Figure 1.**
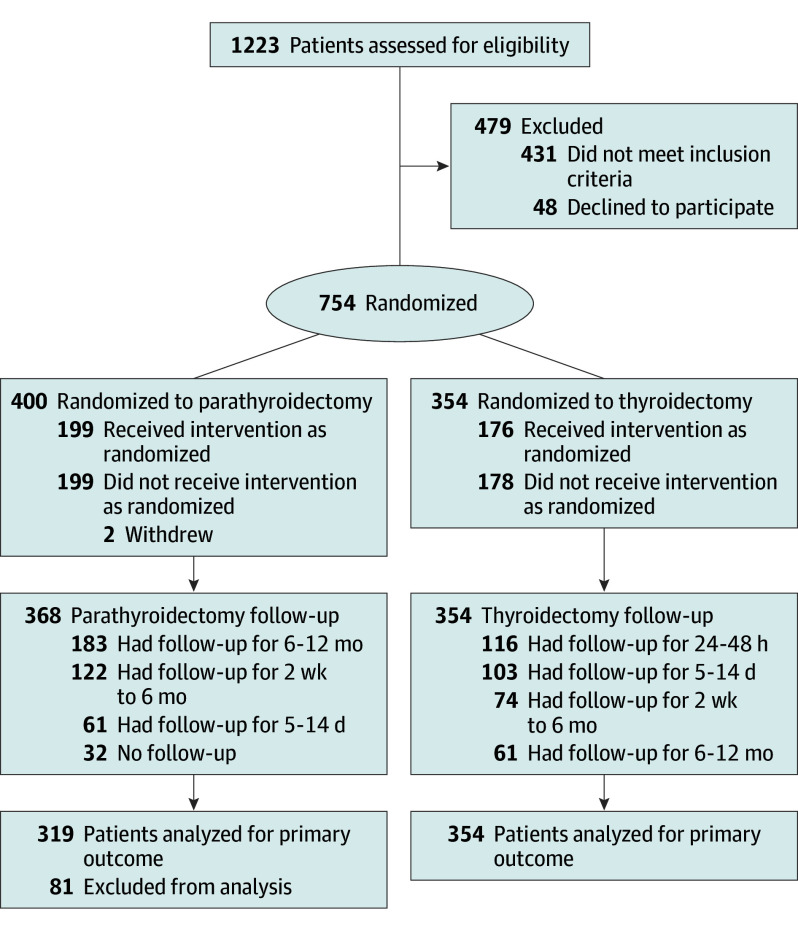
Trial Flowchart

Secondary outcomes include transient (at 24-48 hours after surgery) and last follow-up (beyond 24-48 hours) hypoparathyroidism rates, defined as PTH less than the normal institutional range. After thyroidectomy, follow-up was not required if the initial postoperative PTH was normal and the patient was not taking calcium supplements or activated vitamin D. Real-world follow-up patterns varied (Figure 1). Therefore, the latest PTH was used to determine postsurgical hypoparathyroidism. Other outcomes include operative duration, number of FS (including fine-needle aspirations ), autotransplant rates, and inadvertent PG resection (defined by histology confirmed whole/fragment in thyroid specimen).

Other data collected includes age, sex, race, ethnicity, body mass index, diagnosis, surgical approach, length of stay, laboratory values (PTH, serum calcium, 25-OH vitamin D), and devascularized PGs, as noted by the surgeon. Additionally, for parathyroidectomy, intraoperative PTH drop (more than 50% drop from baseline at 10 minutes postresection) and rate of failure (calcium more than upper limit of normal at the participating center at the last follow-up) were collected.

### Statistical Analysis

Sample size for a 95% powered trial was 33 patients per group using *t* test, calculated using a previous NIRAF-based PG identification study.^34^ Assuming a 30% dropout rate, the trial was designed to enroll 80 patients (40 NIRAF and 40 control) per surgeon per arm, resulting in a target of 400 patients per arm.

Baseline, intraoperative, and postoperative statistics are reported separately for the NIRAF and control groups. Continuous variables are expressed as means and medians with SDs and IQRs and analyzed using the Welch *t* test with their 95% CIs. Categorical variables are presented as frequencies and percentages, and significance assessed via 1-sided χ^2^ or Fisher exact tests. A paired *t* test was used in a subanalysis of the NIRAF group. *P* values <.05 were considered significant. The data were analyzed using R version 4.2.3 (The R Project).

## Results

Overall, 1233 patients underwent screening for eligibility; 754 were randomized, 400 in parathyroidectomy and 354 in the thyroidectomy group and were assigned to the NIRAF or control groups (Figure 1). For the primary outcome, 674 patients were analyzed, 320 parathyroidectomies and 354 thyroidectomies (Figure 1). For parathyroidectomy procedures, 366 patients (92%) had at least 1 follow-up and 183 patients (46%) had follow-up for more than 6 months. All patients had at least 1 follow-up for thyroidectomy procedures and 61 patients (17.2%) had follow-up for more then 6 months. The study groups had balanced baseline characteristics, except for the preoperative PTH levels; the control group had a higher median value (Table 1).

**Table 1.** Baseline Characteristics of Participants

Characteristic	No. (%)					
	Parathyroidectomy			Thyroidectomy		
	Overall (n = 398)	NIRAF (n = 199)	Control (n = 199)	Overall (n = 354)	NIRAF (n = 176)	Control (n = 178)
Age, y, median (IQR)	64 (20)	63 (22)	64 (18)	52 (25)	51.5 (25)	52 (25)
Sex						
Female	304 (76.4)	147 (73.9)	157 (78.9)	267 (75.4)	135 (76.7)	132 (74.2)
Male	94 (23.6)	52 (26.1)	42 (21.1)	87 (24.6)	41 (23.3)	46 (25.8)
Race^a^						
Asian	12 (3.0)	6 (3)	6 (3)	10 (2.8)	5 (2.8)	5 (2.8)
Black	23 (5.8)	10 (5)	13 (6.5)	50 (14.1)	24 (13.6)	26 (14.6)
White	346 (86.9)	176 (88.4)	170 (85.4)	288 (81.4)	143 (81.3)	145 (81.5)
Other^b^	17 (4.3)	7 (3.5)	10 (5)	6 (1.7)	4 (2.3)	2 (1.1)
Ethnicity^a^						
Hispanic	7 (1.8)	2 (1)	5 (2.5)	9 (2.5)	5 (2.8)	4 (2.3)
Non-Hispanic	345 (97.5)	171 (97.2)	174 (97.8)	345 (97.5)	171 (97.2)	174 (97.8)
Multiple or unknown	1 (0.3)	NA	1 (0.5)	NA	NA	NA
Body mass index^c^	27.9 (8.08)	28.2 (8.01)	27.8 (8)	31.1 (10.7)	31.5 (10.22)	30.7 (10.63)
Diagnosis^d^						
Primary sporadic hyperparathyroidism	356 (89.5)	178 (89.5)	178 (89.5)	NA	NA	NA
Recurrent hyperparathyroidism	27 (6.8)	11 (5.5)	16 (8)	NA	NA	NA
Other	15 (3.8)	10 (5)	5 (2.5)	NA	NA	NA
Graves’ disease	NA	NA	NA	99 (28)	51 (29)	48 (27)
Goiters and nodular diseases	NA	NA	NA	110 (31.1)	62 (35.2)	48 (27)
Thyroid cancer	NA	NA	NA	145 (41)	63 (35.8)	82 (46.1)
Preoperative calcium, mg/dL, median (IQR)	10.9 (0.9)	10.9 (0.9)	10.9 (0.8)	9.5 (0.6)	9.5 (0.55)	9.4 (0.6)
Preoperative PTH, pg/mL, median (IQR)	115 (80.75)	115 (84.05)	113 (73)	58.5 (35.52)	52.84 (24.95)	71 (51)
Preoperative vitamin D, ng/mL, median (IQR)	34 (20.15)	33 (20.48)	35 (20)	31.7 (17.4)	33 (17.6)	31.2 (16.55)
Procedure						
Focused	172 (43.2)	86 (43.2)	86 (43.2)	NA	NA	NA
Focused converted to bilateral neck exploration	51 (12.8)	23 (11.6)	28 (14.1)	NA	NA	NA
Bilateral neck exploration (localized)	66 (16.6)	35 (17.6)	31 (15.6)	NA	NA	NA
Bilateral neck exploration (nonlocalized)	104 (26.1)	53 (26.6)	51 (25.6)	NA	NA	NA
Focused converted to unilateral	5 (1.3)	2 (1)	3 (1.5)	NA	NA	NA
Total thyroidectomy	NA	NA	NA	288 (81.4)	147 (83.5)	141 (79.2)
Total thyroidectomy with central neck dissection	NA	NA	NA	46 (13)	21 (11.9)	25 (14)
Patients with prior neck operations	33 (8.3)	13 (6.5)	20 (10.1)	20 (5.7)	8 (4.6)	12 (6.7)

Abbreviations: NA, not applicable; NIRAF, near-infrared autofluorescence; PTH, parathyroid hormone.

SI conversion factors: To convert calcium to mmol/L, multiply by 0.25; to convert PTH to ng/L, multiply by 1; to convert vitamin D to nmol/L, multiply by 2.496.

^a^ Race and ethnicity were self-reported.

^b^ Includes patients who did not self-identify with the racial groups listed (eg, Native American, Pacific Islander, multiracial).

^c^ Calculated as weight in kilograms divided by height in meters squared.

^d^ Refers to low-frequency diagnoses that could not be grouped with the primary diagnoses listed (eg, Hashimoto thyroiditis, multiple endocrine neoplasia type 2, etc).

### Parathyroid Gland Identification With NIRAF

The average number of PGs identified per patient for parathyroidectomy was not significantly different between the NIRAF and control groups during focused procedures (Table 2). However, during bilateral neck explorations, there were more PGs identified across all experience levels, with the NIRAF group exhibiting an average of 3.5 PGs identified per patient (95% CI, 3.4-3.7) and the control group 3.2 PGs per patient (95% CI, 3.0-3.4; *P* < .001). For thyroidectomy, the average number of PGs identified per patient after NIRAF (3.3; 95% CI, 3.2-3.4) was higher than in the control group (2.8; 95% CI, 2.7-3.0; *P* < .001) (Table 2). We also found that the expected number of PGs to be identified increased significantly when using NIRAF during thyroidectomy (eTable in Supplement 1). A subanalysis within the intervention group comparing before vs after NIRAF use shows that during focused parathyroidectomy, the average number of PGs identified by the surgeons did not increase overall after NIRAF (Table 2). However, there was an increase in the number identified during bilateral neck exploration (before NIRAF: 3.1; 95% CI, 2.9-3.3 and after NIRAF: 3.5; 95% CI, 3.4-3.7; *P* < .001). During thyroidectomy, there was an increase in the average number of PGs identified from 2.7 (95% C,: 2.5-2.8) to 3.3 (95% CI, 3.2-3.4; *P* < .001) after using NIRAF (Table 2).

**Table 2.** Average Number of Parathyroid Glands Identified per Patient

Surgeon experience	Intervention (95% CI)		Control (95% CI)	*P* value	
	Before NIRAF	After NIRAF		Before NIRAF vs after NIRAF	Control vs after NIRAF
**Focused parathyroidectomy (n = 112)^a^**					
Overall^b^	1.4 (1.2-1.6)	1.6 (1.4-1.8)	1.5 (1.4-1.7)	.15	.39
Senior (n = 34)	1.3 (1.0-1.6)	1.8 (1.6-2.1)	1.7 (1.5-1.9)	<.05	.33
Junior (n = 78)	1.4 (1.2-1.7)	1.6 (1.3-1.8)	1.4 (1.2-1.6)	.50	.29
Trainee (n = 50)	0.1 (0.04-0.2)	0.7 (0.5-1.0)	NA	<.001	NA
**Bilateral neck exploration parathyroidectomy (n = 207)^b^**					
Overall	3.1 (2.9-3.3)	3.5 (3.4-3.7)	3.2 (3.0-3.4)	<.001	<.01
Senior (n = 126)	3.1 (2.8-3.3)	3.6 (3.4-3.8)	3.3 (3.1-3.5)	<.001	<.05
Junior (n = 81)	3.1 (2.9-3.4)	3.3 (3.0-3.6)	3.0 (2.8-3.3)	.33	.16
Trainee (n = 57)	0.9 (0.6-1.2)	3.1 (2.7-3.5)	NA	<.001	NA
**Thyroidectomy (n = 354)**					
Overall	2.7 (2.5-2.8)	3.3 (3.2-3.4)	2.8 (2.7-3.0)	<.001	<.001
Senior (n = 240)	2.7 (2.4-3.0)	3.3 (3.2-3.5)	2.9 (2.7-3.1)	<.001	<.01
Junior (n = 114)	2.7 (2.4-2.9)	3.2 (3.0-3.5)	2.7 (2.5-2.9)	<.001	<.001
Trainee (n = 104)	1.5 (1.2-1.8)	3.3 (3.1-3.5)	NA	<.001	NA

Abbreviations: NA, not applicable; NIRAF, near-infrared autofluorescence.

^a^ One patient was excluded from the parathyroidectomy data as the confidence data were incomplete (n = 319).

^b^ Trainees were excluded from the overall total confidence metrics because they were not present at every procedure or center, and were under the supervision of an attending surgeon, so they did not contribute to the total number of procedures performed.

### Impact of Surgeon Experience on NIRAF Utility

When analyzing the primary outcome by experience level, the senior surgeons identified more PGs using NIRAF during bilateral neck parathyroid explorations (3.6; 95% CI, 3.4-3.8; *P* < .05), while the junior surgeons did not (Table 2). During thyroidectomy, after using NIRAF, the senior surgeons identified more PGs per patient(3.3; 95% CI, 3.2-3.5). Similarly, the junior surgeons identified more PG per patient (3.2; 95% CI, 3.0-3.5) compared with the control. The subanalysis within the intervention group comparing before and after using NIRAF showed an increase in the number of PGs identified during focused parathyroidectomy for seniors but not juniors (before NIRAF: 1.3; 95% CI, 1.0-1.6 and after NIRAF: 1.8; 95% CI, 1.6-2.1; *P* < .05) (Table 2). During bilateral neck explorations, the senior surgeons increased from an average of 3.1 PGs (95% CI, 2.8-3.3) per patient to 3.6 (95% CI, 3.4-3.8; *P* < .001) after using NIRAF (Table 2). During thyroidectomy, senior surgeons showed increase PG identification from 2.7 (95% CI, 2.4-3.0) to 3.3 (95% CI, 3.2-3.4; *P* < .001) (Table 2). The junior surgeons had more variable results, improving only in the thyroidectomy arm; the rate of PG identification per patient improved from 2.7 (95% CI, 2.4 -2.9) to 3.2 (95% CI, 3.0-2.5; *P* < .001).

Trainee surgeons saw the most significant improvements across both arms of the study. During focused and bilateral neck exploration parathyroidectomy, their identification rate increased from 0.1 (95% CI, 0.04-0.2) to 0.7 (95% CI, 0.5-1.0) PGs per patient and 0.9 (95% CI, 0.6-1.2; *P* < .001) to 3.1 (95% CI, 2.7-3.5; *P* < .001) PGs per patient, respectively. Likewise, during thyroidectomy, the average number of PGs identified by the trainees increased from 1.5 (95% CI, 1.2-1.8) to 3.3 (95% CI, 3.1- 3.5; *P* < .001).

### Distribution of Confidence Levels by Experience

**Figure 2.**
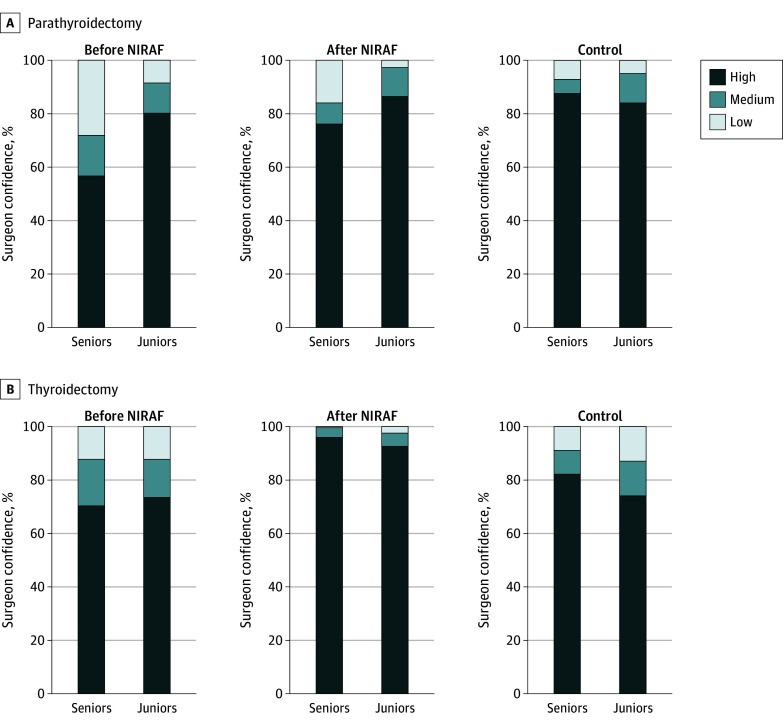
Changes in Surgeon Confidence Surgeon confidence (high >75%, medium >50%, or low <50%) before near-infrared autofluorescence (NIRAF), after NIRAF, and control.

Compared with the senior surgeons, the junior surgeons rated PGs with high confidence more often in both study arms in the control group (Figure 2). Across the study, all surgeons had a lower rate of medium and low confidence in PG identification after using NIRAF. During parathyroidectomy and before NIRAF, the senior surgeons rated 43.3% of PGs with medium or low confidence, while the juniors rated 19.8% with medium or low. After using NIRAF, the senior surgeons rated 23.9% as medium and low, and the juniors only rated 13.6% of PGs with medium and low confidence (Figure 2A). In contrast, during thyroidectomy, senior and junior surgeons had a comparable rate of medium and low confidence PG identification of approximately 30% before NIRAF. After NIRAF, both surgeon groups identified more than 90% of PGs with high confidence (Figure 2B).

### Secondary Outcomes

Data collection for secondary outcomes concluded 6 months after the patient’s initial procedure. There was no difference in the incidence of transient hypoparathyroidism after thyroidectomy between the study groups: 48 in the NIRAF (27.8%; 95% CI, 21.6%-34.8%) and 44 in the control group (26%; 95% CI, 20%-33.1%) (Table 3). At the time of the last follow-up, fewer patients had hypoparathyroidism in the NIRAF group (1.7%; 95% CI, 0.5%-4.9%) than in the control group (4%; 95% CI, 1.9%-8%), but this was not statistically significant.

**Table 3.** Intraoperative and Postoperative Characteristics for Enrolled Patients

Characteristic	Parathyroidectomy arm				Thyroidectomy arm			
	Overall (n = 398)	NIRAF (n = 199)	Control (n = 199)	*P* value	Overall (n = 354)	NIRAF (n = 176)	Control (n = 178)	*P* value
**Intraoperative variables**								
Operative time, min, median (IQR)	80.5 (43.8)	81 (41.5)	78 (48)	.67	123 (53.8)	127.5 (55.3)	120.5 (53.8)	.31
Frozen sections, No. (%)^a^								
0	162 (66.1)	88 (71.5)	74 (60.7)	.10	327 (92.4)	169 (96)	158 (88.8)	<.05
≥1	83 (33.9)	35 (28.5)	48 (39.3)		27 (7.6)	7 (4)	20 (11.2)	
PTH drop^b^	211 (67)	103 (64.4)	108 (69.7)	.32	NA	NA	NA	NA
Parathyroids devascularized, No. (%)								
0	NA	NA	NA	NA	249 (70.3)	119 (67.6)	130 (73)	.27
1	NA	NA	NA		86 (24.3)	49 (27.8)	37 (20.8)	
≥2	NA	NA	NA		19 (5.4)	8 (4.6)	11 (6.2)	
Parathyroids autotransplanted, No. (%)								
0	NA	NA	NA	NA	245 (69.2)	119 (67.6)	126 (70.8)	.72
1	NA	NA	NA		88 (24.9)	47 (26.7)	41 (23)	
≥2	NA	NA	NA		21 (5.9)	10 (5.7)	11 (6.2)	
**Postoperative variables**								
Length of stay								
0 Nights	365 (91.7)	181 (91)	184 (92.5)	.86	365 (91.7)	181 (91)	184 (92.5)	.09
1 Night	17 (4.3)	9 (4.5)	8 (4)		17 (4.3)	9 (4.5)	8 (4)	
>1 Night	16 (4)	9 (4.5)	7 (3.5)		16 (4)	9 (4.5)	7 (3.5)	
Rate of parathyroidectomy failure	12 (3.3)	8 (4.5)	4 (2.1)	.25	NA	NA	NA	NA
Parathyroids inadvertently removed	NA	NA	NA	NA	39 (11)	21 (11.9)	18 (10.1)	.58
Patients with transient hypoparathyroidism	NA	NA	NA	NA	92 (26.9)	48 (27.8)	44 (26)	.72
Patients with hypoparathyroidism at last follow-up	NA	NA	NA	NA	9 (2.6)	3 (1.7)	6 (3.4)	.31

Abbreviations: NA, not applicable; NIRAF, near-infrared autofluorescence; PTH, parathyroid hormone.

^a^ Only bilateral neck explorations and reoperations for parathyroidectomy arm (n = 245).

^b^ One site did not record these values per their standard of care.

Operative times for thyroidectomy were similar between the NIRAF and control groups (median [IQR] time, 127.5 [55.3] minutes vs 120.5 [53.8] minutes). The parathyroidectomy arm had similar results (median [IQR] time, 81 [41.5] minutes vs 78 [48] minutes) (Table 3). In the thyroidectomy arm, there was a decrease in the use of FS analysis in the NIRAF (4%; 95% CI, 1.9%-8%) vs control group (11.2%; 95% CI, 7.4%-16.7%; *P* = .01). During parathyroidectomy, only bilateral explorations and reoperations (n = 245) were included for FS analysis. FS use was lower in the NIRAF (28.5%; 95% CI, 21.3%-37%) vs the control group (39.3%; 95% CI, 31.1%-48.2%), but not statistically significant.

In the thyroidectomy arm, there was no difference in the number of autotransplants performed; 57 patients (32.4%) had at least 1 PG transplanted in the NIRAF and 52 patients in the control group (29.2%). The number of patients who had PGs inadvertently removed was similar in both groups: 21 patients in the NIRAF group (11.9%; 95% CI, 7.9%-17.6%) and 18 in the control group (10.1%; 95% CI, 6.5%-15.4%). In the parathyroidectomy arm, there was a low rate of failure in both groups (8 in the NIRAF group [4.5%; 95% CI, 2.3%-8.6%] and 4 in the control group (2.1%; 95 % CI, 0.8%-5.4%). There was also a low rate of hypoparathyroidism at the last follow-up (3 in the NIRAF group [1.7%] vs 6 in the control group [3.4%]).

## Discussion

The present multicenter randomized clinical trial is the first US-based study describing the efficacy of PG identification using quantitative NIRAF during parathyroidectomies and thyroidectomies. This study finds that fiber-based NIRAF was associated with increased high confidence in PG identification during thyroidectomy and parathyroidectomy and decreased use of FS to confirm PG tissue. Results from previous randomized clinical trials using image-based NIRAF during total thyroidectomy reported increased PG identification, decreased inadvertent PG resection, and possible protection against hypoparathyroidism.^6,8^

This study aimed to assess if fiber-based NIRAF could improve the number of identified PGs intraoperatively. NIRAF increased the average number of PGs identified, similar to other image-based NIRAF studies.^6,8,27,34,35,36,37^ This study showed that NIRAF helped surgeons identify more PGs during thyroidectomy and bilateral neck exploration parathyroidectomy, specifically for senior surgeons and trainees. Similar studies have shown this technology can help bolster the confidence of both residents and attending surgeons.^34,37^ Although it is challenging to measure the impact that more confidently identifying PGs may have on clinical outcomes, NIRAF’s ability to increase confidence during uncertain surgical situations may be crucial to increasing the likelihood of better surgical outcomes.^27^ Additionally, as evidenced by the data, the NIRAF device was an excellent teaching tool for the surgical trainees, providing them with immediate feedback and a boost in confidence as they attempted to identity parathyroid tissue.

The increased ability to identify more PGs during surgery has been associated with decreased postsurgical complications^38,39^ and reduced operative duration,^8^ FS usage,^40,41^ and hospital stay.^42^ However, this work showed no significant difference between the control and intervention groups’ operative duration nor length of stay for either procedural arm, similar to the findings of Bergenfelz et al.^6^ This work showed a decreased use of FS during thyroidectomy, which can reduce hospital operational costs and resource utilization.^43^ While this study did not show decreased FS use during parathyroid operations, the authors’^34^ experience using the device leads them to believe that fiber-based NIRAF can help the surgeon progress through a difficult bilateral neck exploration or reoperative parathyroidectomy, thereby minimizing FS confirmation. Some authors reported that using NIRAF has wholly eliminated FS use during parathyroidectomy. On the other hand, the value of NIRAF during focused parathyroidectomy guided by intraoperative PTH for a single adenoma may be diminished.

Optimizing surgical efficiency is crucial for reducing the risk of postoperative complications arising from longer operative times, failure to identify PGs, and surgeon inexperience.^7,44^ In this study, the surgical sites demonstrated high success rates, with 386 patients (96.7%) having successful procedures. Additionally, 9 thyroidectomy patients (2.6%) had PTH levels less than the institution’s reference range by the last follow-up, indicating a low rate of hypoparathyroidism. None of these metrics differed significantly between the NIRAF and control groups.

The rate of inadvertent PG removal during thyroidectomy was not different between the control and NIRAF in the thyroidectomy arm. This contrasts with the findings of other image-based NIRAF studies that reported a decrease in the intervention group.^6,8,31^ One possible reason for the lack of difference between the 2 groups could be that the study protocol did not require the surgeons to examine each thyroid specimen once it was excised from the neck using NIRAF. As a result, surgeons did not consistently examine specimens with the device for inadvertently resected PGs, leading to a similar number of inadvertently resected PGs being removed in the NIRAF group. Our findings are similar to those of another NIRAF study by Romero-Velez et al,^45^ which only found a decrease in inadvertent PG removal when surgeons used the device to locate PGs in the neck and confirm their absence in the specimen before sending it to pathology. Lastly, another possible difference is that the device does not give a global view of the thyroid. The surgeon needs to scan the entire thyroid with the device (4 mm tip) to avoid missing a PG under the capsule.^46^

It is challenging to determine the impact of NIRAF on preventing postoperative hypoparathyroidism after thyroidectomy due to the overall low rate of adverse events and lack of statistical power in this study. However, 92 of 342 patients (26.9%) had hypoparathyroidism within 24 to 48 hours postoperation and of the 352 patients with complete last follow-up, 9 patients (2.6%) had hypoparathyroidism. There was no difference in hypoparathyroidism rates between the intervention and control groups. Researchers, such as Benmiloud et al,^8^ Bergenfelz et al,^6^ and other randomized clinical trials^37,47^ report similar findings, failing to identify a difference in postoperative hypoparathyroidism between the NIRAF and control groups.

NIRAF demonstrated limited utility in the hands of these surgeons at high-volume centers. Due to the high number of procedures performed annually, junior surgeons at these facilities are more experienced than senior surgeons elsewhere. On the other hand, the trainees demonstrated a substantial increase in confidence in identifying PGs compared with the attending surgeons. As a result of these findings, surgeons who participated in the study were asked to provide feedback on the utility of NIRAF. Collectively, the surgeons confirmed the value of the approach in identifying PGs during uncertain situations, such as nonlocalized parathyroidectomy and in patients with significant scarring or inflammation. NIRAF was particularly valuable during thyroidectomy cases when there was a concurrent lymph node dissection or Hashimoto’s thyroiditis.

### Limitations

The surgeons and centers involved in this study have different surgical approaches and protocols. Therefore, there was no standardized approach, resulting in variations in how the surgeries were performed. We attempted to account for these differences by normalizing the number of PGs expected to be identified, as discussed in the Methods section. These are high-volume centers and surgeons already have highly efficient practices. Therefore, these results may not apply to every surgeon who performs these surgeries. Lastly, this study was designed to determine the impact of NIRAF on the number of PGs identified and not on postoperative conditions, restricting the conclusions that can be drawn.

## Conclusions

The study suggests that during bilateral exploration, fiber-based NIRAF may help surgeons identify more PGs with high confidence but does not provide additional value during focused procedures. During thyroidectomy, the number of PGs identified increased regardless of the procedure. While NIRAF did not significantly impact short-term patient outcomes, it may be a valuable intraoperative tool for surgeons when other methods for PG tissue confirmation are unavailable.

## Notes

Supplement 1. eMethods. Study Schema Background Rationale and Specific Aims Animal Studies and Previous Human Studies Inclusion/Exclusion Criteria Enrollment/Randomization Study Procedures Risks of Investigational Agents/Devices (side effects) Reporting of Adverse Events or Unanticipated Problems involving Risk to Participants or Others Study Withdrawal/Discontinuation Statistical Considerations Privacy/Confidentiality Issues Follow-up and Record Retention eTable. Parathyroid glands indentified by surgeon group and intervention arm

Supplement 2. Data sharing statement
