# Supplementary material for: Near-Infrared Autofluorescence for Parathyroid Detection During Endocrine Neck Surgery: A Randomized Clinical Trial
Source: JAMA Surg. 2025 Jul 16;160(9):936–44. doi: 10.1001/jamasurg.2025.2233 (PMC12268529; doi:10.1001/jamasurg.2025.2233)
Supplement: Supplement 1. — eMethods. Study Schema Background Rationale and Specific Aims Animal Studies and Previous Human Studies Inclusion/Exclusion Criteria Enrollment/Randomization Study Procedures Risks of Investigational Agents/Devices (side effects) Reporting of Adverse Events or Unanticipated Problems involving Risk to Participants or Others Study Withdrawal/Discontinuation Statistical Considerations Privacy/Confidentiality Issues Follow-up and Record Retention eTable. Parathyroid glands indentified by surgeon group and intervention arm [file jamasurg-e252233-s001.pdf]

# Supplemental Online Content

Cousart AG, Kiernan CM, Willmon PA, et al. Near-infrared autofluorescence for parathyroid detection during endocrine neck surgery. *JAMA Surgery*. Published online July 16, 2025. doi:10.1001/jamasurg.2025.2233

**eMethods.** Study Schema
Background
Rationale and Specific Aims
Animal Studies and Previous Human Studies
Inclusion/Exclusion Criteria
Enrollment/Randomization
Study Procedures
Risks of Investigational Agents/Devices (side effects)
Reporting of Adverse Events or Unanticipated Problems involving Risk to Participants or Others
Study Withdrawal/Discontinuation
Statistical Considerations
Privacy/Confidentiality Issues
Follow-up and Record Retention
**eTable**. Parathyroid Glands Identified by Surgeon Group and Intervention Arm

This supplemental material has been provided by the authors to give readers additional information about their work.

## 1.0 Background

Inability of the surgeon to identify or localize the diseased PG can occur in 5 – 10% of cases resulting in failed parathyroidectomies (1, 2). As a result, persistent hyperparathyroidism can occur in these patients resulting in unnecessary repeat surgeries that may be associated with increased morbidity and costs (3, 4). Ultrasound imaging, $^{99m}$Technetium-sestamibi scintigraphy, and computed tomography (CT) have so far demonstrated variable efficacy in preoperative localization of diseased PGs (5, 6) and may not always correlate well with the surgical field of view as observed intraoperatively. Consequently, most surgeons rely on visual identification of PGs during surgery, whereby the accuracy of PG identification is eventually determined by her/his surgical skill and experience (7, 8). When in doubt, a surgeon routinely confirms the identity of PG tissue intraoperatively by sending the specimen for frozen section analysis that typically requires a wait time of 20–30 minutes per sample (9) and has additional costs.

By easily being able to distinguish parathyroid from other tissues intraoperatively, postsurgical complications and associated costs may be reduced. The unique discovery of near infrared autofluorescence (NIRAF) in parathyroid tissues demonstrated that optical modalities that detect NIRAF can be utilized for non-invasive and label-free identification of parathyroid tissues with an accuracy as high as 97%.(10, 11) Since then, several research groups have explored the feasibility of localizing parathyroid glands using NIRAF detection with reasonable success, resulting in FDA clearance for marketing this optical technique (12). In this study, we plan to evaluate whether an FDA-cleared device called 'PTeye' (AiBiomed, Santa Barbara, CA) is beneficial or not, for the surgeon and patient during parathyroid operations. The results of such a study will help us to understand and assess the true impact of optical modalities such as PTeye on (i) improving the quality and efficiency of parathyroid surgeries and (ii) minimizing risk of postsurgical complications and related expenses.

## 2.0 Rationale and Specific Aims

The goal of this study is to assess whether using PTeye – a NIRAF detection modality – can improve patient outcomes and reduce healthcare associated costs after parathyroid surgeries. The specific aims of this study is to determine if PTeye is beneficial or not for (i) intraoperative identification of parathyroid tissues, (ii) improving efficiency of parathyroid surgeries, and (iii) minimizing risk of postsurgical complications.

By being able to quickly and definitively locate parathyroid glands while in the operating room, the duration of surgical procedure could be further reduced. In addition, the number of frozen section biopsy and associated costs can be minimized. Furthermore, repeat surgeries as a result of missing a diseased parathyroid gland at the time of the initial parathyroidectomy for hyperparathyroidism could potentially be avoided.

## 3.0 Animal Studies and Previous Human Studies

Modalities that rely on NIRAF detection for label-free parathyroid identification have been successfully validated in several studies (13-16). FDA clearance for marketing this application was recently granted to Fluobeam (a commercially available imaging system) and PTeye (a commercial fiber probe-based system) in 2018 (12). Certain outcome studies have reported that imaging-based systems for NIRAF detection such as the PDE Neo II system was able to reduce the number of frozen sections required during

parathyroid procedures (15). However, other studies have reported that they observed no benefit from imaging-based systems in parathyroid localization (17). In a recent study, Thomas *et al.* demonstrated that a fiber probe-based system – the PTeye – was more sensitive in parathyroid identification compared to the imaging-based system by PDE Neo II (18). To date, there has been no studies that determine the impact of a fiber probe-based system (i.e. PTeye) during parathyroid surgeries in minimizing a number of frozen sections obtained intraoperatively or postsurgical complications.

## 4.0 Inclusion/Exclusion Criteria

Inclusions:

- All adults (i.e., ≥ 18 years old) patients with primary hyperparathyroidism who will be undergoing parathyroid surgery
- All adults (i.e., ≥ 18 years old) patients with persistent primary hyperparathyroidism after having undergone a failed prior parathyroid surgery who will be undergoing repeat parathyroid surgery

Exclusion:

- Children and minors
- Pregnant women
- Patients with concurrent parathyroid and thyroid disease that require total thyroidectomy
- Patients with secondary or tertiary hyperparathyroidism

## 5.0 Enrollment/Randomization

The research will be designed as a single center study, where patient recruitment will be conducted by the participating surgeons at Vanderbilt University Medical Center (VUMC). At VUMC, the initial evaluation will be conducted while the surgeon is evaluating the patients at the Endocrine Surgery Clinics at the Vanderbilt Eskind Diabetes Center, Vanderbilt Ingram Cancer Center or the Odess clinic (for ENT patients). The final eligibility of each patient for participating in this study will be determined by the participating surgeon in accordance of his/her medical conditions. Following the exclusion criteria, the study will aim to accrue all eligible patients who are going to the OR as part of their standard surgical procedure:

1. On the date of procedure consent at clinic or the date of surgery, patients will be given a paper consent form detailing about the outcome study with PTeye in addition to the consent for the surgical procedure that will be performed.
2. The key study personnel will briefly describe the PTeye and its application to the patient.
3. If the patient is interested in participating, the key study personnel will provide the patient with a paper-based consent form.
4. The key study personnel or the surgeon assigned to the patient will be available to discuss the protocol with the patient (including, risks, benefits, alternatives, etc.). The patient will also be provided with the contact information of the principal investigator should they have any questions.
5. If the patient consents to be part of the study, a scan of their signed consent form will be stored digitally in the patient’s medical record.

Prior to surgery, the patient will be assigned a unique ID (e.g. Para_001). The unique ID will be utilized for randomly allocating the patient to the experimental arm (where the surgeon will use PTeye) or a control arm (where the surgeon will not use PTeye). The allocation will be conducted by the study coordinator using 'Random Allocation Software' (http://mahmoodsaghaei.tripod.com/Softwares/randalloc.html).

## 6.0 Study Procedures

For patients assigned to the study arm, the surgeon will use the PTeye as an intraoperative tool to identify if a suspect tissue is a parathyroid or not, during the parathyroid surgery. The surgeon will first take 5 baseline NIRAF measurements on the thyroid gland (or neck muscle, if thyroid is absent) using the disposable sterile fiber probe that is connected to the PTeye console (see Figure 1), as per device functionality requirements. The subsequent step would involve touching the target tissue in the neck with the fiber optic probe, following which the PTeye will indicate to the surgeon if the tissue is parathyroid or not. Parameters which are displayed on the PTeye console – Baseline values, Detection Intensity, Parathyroid Detection Ratio – will be recorded for each patient enrolled in the study arm. PTeye indicates that a tissue is parathyroid if it displays the 'Parathyroid Detection Ratio' is greater than 1.2. The rest of the surgical procedure will follow according to standard protocol.

For patients assigned to the control arm, the surgeon will not use the PTeye and will proceed with the parathyroid surgery as usual, while relying solely on her/his surgical experience in identifying the parathyroid glands during the operations.

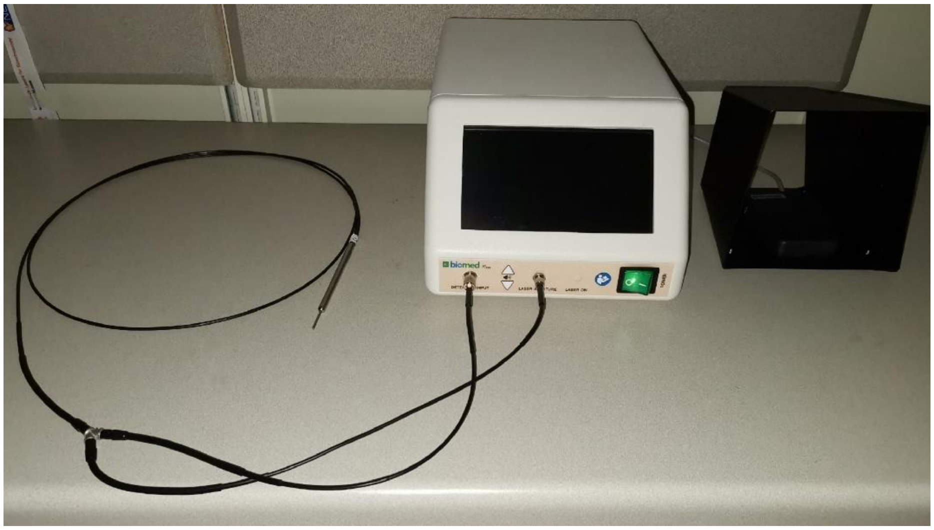

**Figure 1.** A commercial fiber probe-based system (PTeye, AiBiomed) for detecting near infrared autofluorescence (NIRAF) utilized for intraoperative parathyroid gland identification. PTeye consists of 1) the console that has a display and indicates to the surgeon if a tissue is parathyroid or not, 2) a detachable fiber optic probe, and 3) a foot-pedal which is activated by the surgeon for tissue NIRAF measurements.

De-identified information regarding (i) patient demographics, (ii) duration of surgery, (iii) number of frozen section analysis performed, (iv) frozen section and permanent histology reports of all excised tissues, (v) blood calcium levels (before surgery, 1st postoperative

visit after surgery and 6 months after surgery (if followed up)), (vi) parathyroid hormone (PTH) levels (1st postoperative visit after surgery and 6 months after surgery (if followed up)) and (vii) postsurgical complications, if any and history of ER visits or hospitalization or repeat surgeries due to high calcium, will be compiled for all enrolled patients. These preoperative, intraoperative and postoperative investigations are to be performed at the surgeon's discretion as necessary for the patient. These parameters would then be compared between the study arm and the control arm to truly gauge the impact or value of a device like PTeye for parathyroid surgeries.

## 7.0 Risks

- The proposed study is designed to collect NIRAF measurements from neck tissues with a commercial device called PTeye during a parathyroidectomy..
- Each PTeye measurement takes less than 2 seconds, with the whole set of measurements not adding more than 5 minutes to the surgical procedure. Thus, there is a minimal increase of risk of surgery due to the potential five extra minutes of anesthesia time associated with the study. In addition, the participating surgeon will evaluate the eligibility of the patient based on his or her medical condition. Patients with high anesthetic risks will not be asked to participate in the study.

- Since the power of near infrared light from PTeye will be extremely low, no side effects should be introduced to the patient.

- There should not be any discomforts, inconveniences, and/or risk resulting from this study.

- The study should not increase the risk of infection as a disposable sterile probe is used for each patient.

- The PTeye device used in this study has been granted clearance for marketing by the FDA. The marketing authorization by FDA has been granted based on the caveat that necessary precautionary measures will be taken by the surgeon to minimize the probable risks (as listed in the device brochure). The PTeye may be associated with unknown/unforeseen risks as with any other FDA-cleared medical devices used during surgical procedures.

## 8.0 Reporting of Adverse Events or Unanticipated Problems involving Risk to Participants or Others

Upon identification, adverse events resulting from this *in vivo* data acquisition procedure will be reported to the PI as well as the Vanderbilt IRB immediately. The study will be immediately terminated and not resumed until the sources leading to the adverse events are identified.

## 9.0 Study Withdrawal/Discontinuation

The data acquisition procedure will be terminated if the medical conditions of the participant show some unexpected and adverse changes. The decision of terminating the study will be determined by the participating surgeon.

## 10.0 Statistical Considerations

Benmiloud *et al*. studied the impact of NIRAF-based identification of parathyroid glands which comprised of a group of 93 patients where NIRAF was used by a surgeon for parathyroid identification and a control group of 153 patients where NIRAF was not used (19). Their study reported that mean parathyroid glands identified by surgeon with NIRAF imaging was significantly higher at 3.1±0.9, while that of same surgeon without NIRAF imaging was 2.6±0.1 (p=0.0001). Based on this data (mean difference: 0.5 and standard deviation: 1.0), it was determined that in order to observe a statistically significant difference (i.e. for an expected mean difference: 0.7 and expected standard deviation:1.0), 33 patients would be required per group (for a 95% powered study). Since this study may involve patient follow-up for data up to 6 months after surgery, we will thus assume an approximate data attrition rate of 20%, thus requiring a recruitment of 40 patients per group. Therefore, a total of 80 patients (study and control arm) should be recruited per surgeon for this study at this study site. To assess the influence of impact for surgeons based on her/his surgical experience, one senior surgeon (>10 years of experience) and one junior surgeon (<5 years of experience) will participate in study. Overall, 160 patients need to be enrolled to assess the impact of this technology.

## 11.0 Privacy/Confidentiality Issues

All study staff are required to complete the CITI Protection of Human Subjects Training Program. All data pertaining to this study will be stored in the HIPPA compliant REDcap data management program. Access to study data will be limited to the investigators listed on the study only. Only indirect identifiers such as medical record number (MRN) will be used. Participants' information, including his or her name and medical record number, will be available only to the PI and key study personnel. It will be stored in a password-protected computer and a locked file cabinet in the study coordinator's office where we also will store the consent forms. All records will only be available to the PI and key study personnel. All information potentially identifying the participant will not be included in the data analysis or reporting of results therein.

## 12.0 Follow-up and Record Retention

The duration of the study is expected to be approximately two year and will depend on patient availability. The data acquired from this study will be preserved indefinitely, as it may influence the future development of the entire research project. However, the data will not be accessible to anyone other than the participants of this study. All original paper records, record sheets, preoperative and postoperative lab investigations, drug/medication history, post-surgical medical history, histopathological diagnoses of the investigated tissue samples, will be collectively retained by the PI or Key Study Personnel. The data of this study will be stored in a password protected computer, and only users with permission from the PI can access the data base.

## 13.0 eReferences


1. Simental A, Ferris RL. Reoperative Parathyroidectomy. Otolaryngologic Clinics of North America. 2008 2008/12/01/;41(6):1269-74.
2. Cron DC, Kapeles SR, Andraska EA, et al. Predictors of operative failure in parathyroidectomy for primary hyperparathyroidism. The American Journal of Surgery. 2017;214:509-14.
3. Doherty GM, Weber B, Norton JA. Cost of unsuccessful surgery for primary hyperparathyroidism. Surgery. 1994;116(6):954-8.
4. Wachtel H, Cerullo I, Bartlett EK, et al. What Can We Learn from Intraoperative Parathyroid Hormone Levels that Do Not Drop Appropriately? Annals of Surgical Oncology. 2015 June 01;22(6):1781-8.
5. Mohebati A, Shaha AR. Imaging techniques in parathyroid surgery for primary hyperparathyroidism. American Journal of Otolaryngology. 2012 12/07;33(4):457-68.
6. Ahuja AT, Wong KT, Ching ASC, et al. Imaging for primary hyperparathyroidism — what beginners should know. Clinical Radiology. 2004 11//;59(11):967-76.
7. Sosa JA, Powe NR, Levine MA, et al. Thresholds for Surgery and Surgical Outcomes for Patients with Primary Hyperparathyroidism: A National Survey of Endocrine Surgeons. The Journal of Clinical Endocrinology & Metabolism. 1998;83(8):2658-65.
8. Chen H, Wang TS, Yen TWF, et al. Operative Failures after Parathyroidectomy for Hyperparathyroidism: The Influence of Surgical Volume. Annals of Surgery. 2010;252(4):691-5.
9. Novis DA, Zarbo RJ. Interinstitutional comparison of frozen section turnaround time. Archives of pathology & laboratory medicine. 1997;121(6):559.
10. McWade MA, Sanders ME, Broome JT, et al. Establishing the clinical utility of autofluorescence spectroscopy for parathyroid detection. Surgery. 2016 1//;159(1):193-203.
11. McWade MA, Paras C, White LM, et al. A novel optical approach to intraoperative detection of parathyroid glands. Surgery. 2013 12//;154(6):1371-7.
12. The United States Food and Drug Administration. FDA permits marketing of two devices that detect parathyroid tissue in real-time during surgery. Available at: https://www.fda.gov/NewsEvents/Newsroom/PressAnnouncements/ucm624982.htm. Accessed November 3, 2018.
13. Ladurner R, Sommerey S, Arabi NA, et al. Intraoperative near-infrared autofluorescence imaging of parathyroid glands. Surgical Endoscopy. 2016:1-6
14. Falco J, Dip F, Quadri P, et al. Increased identification of parathyroid glands using near infrared light during thyroid and parathyroid surgery. Surgical Endoscopy. 2017 September 01;31(9):3737-42.
15. Squires MH, Jarvis R, Shirley LA, Phay JE. Intraoperative Parathyroid Autofluorescence Detection in Patients with Primary Hyperparathyroidism. Annals of Surgical Oncology. 2019;26:1142-8.
16. Thomas G, McWade MA, Paras C, et al. Developing a clinical prototype to guide surgeons for intraoperative label-free identification of parathyroid glands in real time. Thyroid. 2018;28(11):1517-31
17. DiMarco A, Chotalia R, Bloxham R, et al. Autofluorescence in Parathyroidectomy: Signal Intensity Correlates with Serum Calcium and Parathyroid Hormone but Routine Clinical Use is Not Justified. World journal of surgery. 2019 Jun 15;43(6):1532-7.

18. Thomas G, Squires MH, Metcalf, T., et al. Imaging or a Fiber Probe-based Approach? Assessing Different Methods to Detect Near Infrared Autofluorescence for Intraoperative Parathyroid Identification. Journal of the American College of Surgeons. 2019; (in press)
19. Benmiloud F, Rebaudet S, Varoquaux A, et al. Impact of autofluorescence-based identification of parathyroids during total thyroidectomy on postoperative hypocalcemia: a before and after controlled study. Surgery. 2018; 163(1):23-30

# Assessing Benefits of Near Infrared Autofluorescence (NIRAF) Detection for Identifying Parathyroid Glands during Total Thyroidectomy.

*Research Protocol*

**Carmen C. Solórzano, MD, FACS**
Professor of Surgery
Chair, Department of Surgery
Director, Vanderbilt Endocrine Surgery
Vanderbilt University Medical Center

**Colleen M. Kiernan, MD, MPH, FACS**
Assistant Professor of Surgery
Department of Surgery

Version date 07/26/2022

**Table of Contents:**

**Study Schema**
**1.0 Background**
**2.0 Rationale and Specific Aims**
**3.0 Animal Studies and Previous Human Studies**
**4.0 Inclusion/Exclusion Criteria**
**5.0 Enrollment/Randomization**
**6.0 Study Procedures**
**7.0 Risks of Investigational Agents/Devices (side effects)**
**8.0 Reporting of Adverse Events or Unanticipated Problems involving Risk to Participants or Others**
**9.0 Study Withdrawal/Discontinuation**
**10.0 Statistical Considerations**
**11.0 Privacy/Confidentiality Issues**
**12.0 Follow-up and Record Retention**

## 14.0 Background

Inadvertent damage to or excision of a healthy parathyroid gland (PG) following a total thyroidectomy could result in transient hypocalcemia (< 6 months) in 5 – 35% of cases or permanent hypocalcemia (> 6 months) in up to 7% of the patients (1, 2). Patient that develop hypocalcemia will thus need to take high doses of oral calcium and vitamin D levels every 4–8 hours temporarily if it is a case of transient hypocalcemia or for the rest of their life if is a case of permanent hypocalcemia. Preoperative modalities such as ultrasound imaging, $^{99m}$Technetium-sestamibi scintigraphy, and computed tomography (CT) are not as effective in localizing healthy parathyroid glands and may not always correlate well with the surgical field of view as observed intraoperatively. Consequently, most surgeons rely on visual identification of PGs during surgery, whereby the accuracy of PG identification is eventually determined by her/his surgical skill and experience (3). When in doubt, a surgeon routinely confirms the identity of PG tissue intraoperatively by sending the specimen for frozen section analysis that typically requires a wait time of 20–30 minutes per sample (4), is costly and has risk of possible injury to a healthy PG.

By easily being able to distinguish parathyroid from other tissues intraoperatively, postsurgical complications and associated costs may be reduced. The unique discovery of near infrared autofluorescence (NIRAF) in parathyroid tissues demonstrated that optical modalities that detect NIRAF can be utilized for non-invasive and label-free identification of parathyroid tissues with an accuracy as high as 97% (5, 6). Since then, several research groups have explored the feasibility of localizing parathyroid glands using NIRAF detection with reasonable success, resulting in FDA permitting marketing for this optical technique (7). In this study, we plan to evaluate whether the device called 'PTeye' (AiBiomed, Santa Barbara, CA) is beneficial or not, for the surgeon and patient during total thyroidectomies. The results of such a study will help us to understand and assess the true impact of optical modalities such as PTeye on (i) improving the quality and efficiency of total thyroidectomies and (ii) minimizing risk of postsurgical complications and related expenses.

## 15.0 Rationale and Specific Aims

The goal of this study is to assess whether using PTeye – a NIRAF detection modality – can improve patient outcomes and reduce healthcare associated costs after total thyroidectomy procedures. The specific aims of this study is to determine if PTeye is beneficial or not for (i) intraoperative identification of parathyroid tissues, (ii) improving efficiency of total thyroidectomies, and (iii) minimizing risk of postsurgical complications.

By being able to quickly and definitively locate parathyroid glands while in the operating room, the duration of surgical procedure could be further reduced. In addition, the number of frozen section biopsy and associated costs can be minimized. Furthermore, accidental excision or damage to healthy parathyroid glands during total thyroidectomy procedures can be decreased, thus minimizing the risk for postoperative hypocalcemia and hypoparathyroidism.

## 16.0 Animal Studies and Previous Human Studies

Modalities that rely on NIRAF detection for label-free parathyroid identification have been successfully validated in several studies (8-11). FDA recently allowed marketing to Fluobeam (a commercially available imaging system) and PTeye (a commercial fiber

probe-based system) in 2018 (7). Certain outcome studies have reported that imaging-based systems for NIRAF detection such as Fluobeam was able to reduce the incidence of postsurgical transient hypocalcemia after total thyroidectomy (10, 11). However, other studies have reported that they observed no benefit from imaging-based systems in minimizing postsurgical hypocalcemia (13). In a recent study, Thomas *et al.* demonstrated that a fiber probe-based system – the PTeye – was more sensitive in parathyroid identification compared to an imaging-based system for NIRAF detection (14). To date, there has been no studies that determine the impact of a fiber probe-based system (i.e. PTeye) during total thyroidectomy operations in minimizing a number of frozen sections obtained intraoperatively or postsurgical complications.

## 17.0 Inclusion/Exclusion Criteria

Inclusions:

- All adults (i.e., ≥ 18 years old) patients with thyroid disease who will be undergoing total thyroidectomy (includes patients who have undergone a prior neck exploration for parathyroid disease or other but have an intact thyroid gland).
- All adults (i.e., ≥ 18 years old) patients with persisting thyroid disease and will be undergoing re-operative or completion thyroidectomy.

Exclusion:

- Children and minors
- Pregnant women
- Patients with concurrent parathyroid and thyroid disease
- Patients with incidental enlarged parathyroid discovered during thyroidectomy procedure.
- Patients undergoing thyroid lobectomy or partial thyroidectomy

## 18.0 Enrollment/Randomization

The research will be designed as a single center study, where patient recruitment will be conducted by the participating surgeons at Vanderbilt University Medical Center (VUMC). At VUMC, the initial evaluation will be conducted while the surgeon is evaluating the patients at the Endocrine Surgery Clinics at the Vanderbilt Eskind Diabetes Center, Vanderbilt Ingram Cancer Center or the Odess clinic (for ENT patients). The final eligibility of each patient for participating in this study will be determined by the participating surgeon in accordance of his/her medical conditions. Following the exclusion criteria, the study will aim to accrue all eligible patients who are going to the OR as part of their standard surgical procedure:

1. On the date of procedure consent at clinic or the date of surgery, patients will be given a paper consent form detailing about the outcome study with PTeye in addition to the consent for the surgical procedure that will be performed.
2. The key study personnel will briefly describe the PTeye and its application to the patient.
3. If the patient is interested in participating, the key study personnel will provide the patient with a paper-based consent form.
4. The key study personnel or the surgeon assigned to the patient will be available to discuss the protocol with the patient (including, risks, benefits, alternatives, etc.).

The patient will also be provided with the contact information of the principle investigator should they have any questions.

5. If the patient consents to be part of the study, a scan of their signed consent form will be stored digitally in the patient's medical record.

Prior to surgery, the patient will be assigned a unique ID (e.g. TThy_001). The unique ID will be utilized for randomly allocating the patient to the experimental arm (where the surgeon will use PTeye) or a control arm (where the surgeon will not use PTeye). The allocation will be conducted by the study coordinator using 'Random Allocation Software' (http://mahmoodsaghaei.tripod.com/Softwares/randalloc.html).

## 19.0 Study Procedures

For patients assigned to the study arm, the surgeon will use the PTeye as an intraoperative tool to identify if a suspect tissue is a parathyroid or not, during the total thyroidectomy procedure. The surgeon will first take 5 baseline NIRAF measurements on the thyroid gland (or neck muscle, if thyroid is absent) using the disposable sterile fiber probe that is connected to the PTeye console (see Figure 1), as per device functionality requirements. The subsequent step would involve touching the target tissue in the neck with the fiber optic probe, following which the PTeye will indicate to the surgeon if the tissue is parathyroid or not. Parameters which are displayed on the PTeye console – Baseline values, Detection Intensity, Parathyroid Detection Ratio – will be recorded for each patient enrolled in the study arm. PTeye indicates that a tissue is parathyroid if it displays the 'Parathyroid Detection Ratio' is greater than 1.2. The rest of the surgical procedure will follow according to standard protocol.

For patients assigned to the control arm, the surgeon will not use the PTeye and will proceed with the total thyroidectomy as usual, while relying solely on her/his surgical experience in identifying the parathyroid glands during the operations.

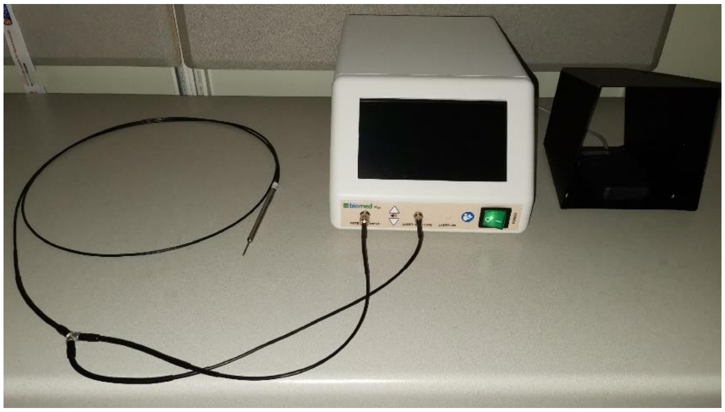

**Figure 1.** A commercial fiber probe-based system (PTeye, AiBiomed) for detecting near infrared autofluorescence (NIRAF) utilized for intraoperative parathyroid gland identification. PTeye consists of 1) the console that has a display and indicates to the surgeon if a tissue is parathyroid or not, 2) a detachable fiber optic probe, and 3) a foot-pedal which is activated by the surgeon for tissue NIRAF measurements.

De-identified information regarding (i) patient demographics, (ii) duration of surgery, (iii) number of frozen section analysis performed, (iv) frozen section and permanent histology reports of all excised tissues, (v) blood calcium levels (before surgery, within 24 hours

after surgery, 1st postoperative visit after surgery and 6 months after surgery (if followed up)), (vi) blood parathyroid hormone (PTH) levels (within 24 hours after surgery, 1st postoperative visit after surgery and 6 months after surgery (if followed up), (vii) history and duration of calcium and/or Vitamin D supplementation before and after surgery and (viii) postsurgical complications, if any and history of ER visits or hospitalization due to low blood calcium will be compiled for all enrolled patients. These preoperative, intraoperative and postoperative investigations are to be performed at the surgeon's discretion as necessary for the patient. These parameters would then be compared between the study arm and the control arm to truly gauge the impact or value of a device like PTeye for total thyroidectomies.

## 20.0 Risks

- The proposed study is designed to collect NIRAF measurements from neck tissues with a commercial device called PTeye during a total thyroidectomy. The device that will be used for NIRAF measurements has been permitted for marketing by the FDA.
- Each PTeye measurement takes less than 2 seconds, with the whole set of measurements not adding more than 5 minutes to the surgical procedure. Thus, there is a minimal increase of risk of surgery due to the potential five extra minutes of anesthesia time associated with the study. In addition, the participating surgeon will evaluate the eligibility of the patient based on his or her medical condition. Patients with high anesthetic risks will not be asked to participate in the study.
- Since the power of near infrared light from PTeye will be extremely low, no side effects should be introduced to the patient.
- There should not be any discomforts, inconveniences, and/or risk resulting from this study.
- The study should not increase the risk of infection as a disposable sterile probe is used for each patient.
- The PTeye device used in this study has been permitted by the FDA for marketing. The FDA permit for marketing is granted based on the caveat that necessary precautionary measures will be taken by the surgeon to minimize the probable risks (as listed in the device brochure). The PTeye may be associated with unknown/unforeseen risks as with any other FDA cleared for marketing medical devices used during surgical procedures.

## 21.0 Reporting of Adverse Events or Unanticipated Problems involving Risk to Participants or Others

Upon identification, adverse events resulting from this *in vivo* data acquisition procedure will be reported to the PI as well as the Vanderbilt IRB immediately. The study will be immediately terminated and not resumed until the sources leading to the adverse events are identified.

## Study Withdrawal/Discontinuation

The data acquisition procedure will be terminated if the medical conditions of the participant show some unexpected and adverse changes. The decision of terminating the study will be determined by the participating surgeon.

## 22.0 Statistical Considerations

Benmiloud *et al*. studied the impact of NIRAF-based identification of parathyroid glands during total thyroidectomies, which comprised of a group of 93 patients where NIRAF was used by a surgeon for parathyroid identification and a control group of 153 patients where NIRAF was not used (10). Their study reported that mean parathyroid glands identified by surgeon with NIRAF imaging was significantly higher at 3.1±0.9, while that of same surgeon without NIRAF imaging was 2.6±0.1 (p=0.0001). Based on this data (mean difference: 0.5 and standard deviation: 1.0), it was determined that in order to observe a statistically significant difference (i.e. for an expected mean difference: 0.7 and expected standard deviation: 1.0), 33 patients would be required per group (for a 95% powered study). Since this study may involve patient follow-up for data up to 6 months after surgery, we will thus assume an approximate data attrition rate of 20%, thus requiring a recruitment of 40 patients per group. Therefore, a total of 80 patients (study and control arm) should be recruited per surgeon for this study at this site. To assess the influence of impact for surgeons based on her/his surgical experience, one senior surgeon (>10 years of experience) and one junior surgeon (<5 years of experience) will participate in study. Overall, 160 patients need to be enrolled to assess the impact of this technology.

## 23.0 Privacy/Confidentiality Issues

All study staff are required to complete the CITI Protection of Human Subjects Training Program. All data pertaining to this study will be stored in a HIPPA-compliant data spread-sheet with access limited solely to the investigators listed on the study. Only indirect identifiers such as medical record number (MRN) will be used. Participants' information, including his or her name and medical record number, will be available only to the PI and key study personnel. It will be stored in a password-protected computer and a locked file cabinet in the study coordinator's office where we also will store the consent forms. All records will only be available to the PI and key study personnel. All information potentially identifying the participant will not be included in the data analysis or reporting of results therein.

## 24.0 Follow-up and Record Retention

The duration of the study is expected to be approximately two year and will depend on patient availability. The data acquired from this study will be preserved indefinitely, as it may influence the future development of the entire research project. However, the data will not be accessible to anyone other than the participants of this study. All original paper records, record sheets, preoperative and postoperative lab investigations, drug/medication history, post-surgical medical history, histopathological diagnoses of the investigated tissue samples, will be collectively retained by the PI or Key Study Personnel. The data of this study will be stored in a password protected computer, and only users with permission from the PI can access the data base.

## 25.0 eReferences

1. Antakia R, Edafe O, Uttley L, Balasubramanian SP. Effectiveness of Preventative and Other Surgical Measures on Hypocalcemia Following Bilateral Thyroid Surgery: A Systematic Review and Meta-Analysis. Thyroid. 2014; 25:95-106.
2. Edafe O, Antakia R, Laskar N, et al. Systematic review and meta-analysis of predictors of post-thyroidectomy hypocalcaemia. The British journal of surgery. 2014 Mar; 101(4):307-20.
3. Sosa JA, Bowman HM, Tielsch JM, et al. The Importance of Surgeon Experience for Clinical and Economic Outcomes from Thyroidectomy. Annals of Surgery. 1998; 228(3):320-30.
4. Novis DA, Zarbo RJ. Interinstitutional comparison of frozen section turnaround time. Archives of pathology & laboratory medicine. 1997; 121(6):559.
5. McWade MA, Sanders ME, Broome JT, et al. Establishing the clinical utility of autofluorescence spectroscopy for parathyroid detection. Surgery. 2016; 159(1):193-203.
6. McWade MA, Paras C, White LM, et al. A novel optical approach to intraoperative detection of parathyroid glands. Surgery. 2013 12//; 154(6):1371-7.
7. The United States Food and Drug Administration. FDA permits marketing of two devices that detect parathyroid tissue in real-time during surgery. Available at: https://www.fda.gov/NewsEvents/Newsroom/PressAnnouncements/ucm624982.htm. Accessed November 3, 2018.
8. Ladurner R, Sommerey S, Arabi NA, et al. Intraoperative near-infrared autofluorescence imaging of parathyroid glands. Surgical Endoscopy. 2016:1-6
9. Falco J, Dip F, Quadri P, et al. Increased identification of parathyroid glands using near infrared light during thyroid and parathyroid surgery. Surgical Endoscopy. 2017 September 01; 31(9):3737-42.
10. Benmiloud F, Rebaudet S, Varoquaux A, et al. Impact of autofluorescence-based identification of parathyroids during total thyroidectomy on postoperative hypocalcemia: a before and after controlled study. Surgery. 2018; 163(1):23-30
11. Benmiloud F, Godiris-Petit G, Gras R, et al. Association of Autofluorescence-Based Detection of the Parathyroid Glands During Total Thyroidectomy With Postoperative Hypocalcemia Risk: Results of the PARAFLUO Multicenter Randomized Clinical Trial. JAMA Surgery. 2019; (in press)
12. Thomas G, McWade MA, Paras C, et al. Developing a clinical prototype to guide surgeons for intraoperative label-free identification of parathyroid glands in real time. Thyroid. 2018; 28(11):1517-31
13. DiMarco A, Chotalia R, Bloxham R, et al. Does fluoroscopy prevent inadvertent parathyroidectomy in thyroid surgery? The Annals of the Royal College of Surgeons of England. 2019 Jul 15; 101(7):508-13.
14. Thomas G, Squires MH, Metcalf, T., et al. Imaging or a Fiber Probe-based Approach? Assessing Different Methods to Detect Near Infrared Autofluorescence for Intraoperative Parathyroid Identification. Journal of the American College of Surgeons. 2019; (in press)

**eTable**. Parathyroid Glands Identified by Surgeon Group and Intervention Arm

The total number of parathyroid glands (PGs) identified per patient with and without Near Infrared Autofluorescence (NIRAF) was normalized to the expected number of PGs (4 during total thyroidectomy and 2 during completion thyroidectomy) to adjust for surgeon and procedural differences. Focused procedures (1 PG), unilateral neck exploration (2 PGs), and bilateral neck explorations (4 PGs) were normalized accordingly for parathyroidectomy. Trainees only participated in the intervention group.

Parathyroid Glands Identified by Surgeon Group and Intervention Arm

| **Surgeon Group** | **Number of PGs identified per patient / the expected number of PGs identified** | | | | |
|---|---|---|---|---|---|
| | **Intervention (95% CI)** | | **Control (95% CI)** | **Before NIRAF vs After** | **Control vs After NIRAF** |
| | **Before NIRAF** | **After NIRAF** | | *P* | *P* |
| **Parathyroidectomy** | | | | | |
| Overall | 0.86 (0.79 - 0.92) | 0.97 (0.91 - 1.0) | 0.88 (0.79 - 0.97) | **<.01** | .11 |
| Seniors | 0.79 (0.72 - 0.87) | 0.96 (0.9 - 1.0) | 0.92 (0.8 - 1.0) | **<.001** | .55 |
| Juniors | 0.92 (0.82 - 1.0) | 0.98 (0.88 - 1.1) | 0.85 (0.71 - 0.99) | .44 | .12 |
| Trainees | 0.14 (0.1 - 0.2) | 0.51 (0.42 - 0.6) | - | **<.001** | - |
| **Thyroidectomy** | | | | | |
| Overall | 0.78 (0.71 - 0.84) | 0.96 (0.90 - 1.02) | 0.83 (0.77 - 0.89) | **< .001** | **<.01** |
| Seniors | 0.81 (0.72 - 0.89) | 1.0 (0.93 - 1.1) | 0.87 (0.79 - 0.95) | **< .001** | **<.05** |
| Juniors | 0.71 (0.63 - 0.78) | 0.85 (0.77 - 0.92) | 0.74 (0.67 - 0.81 | **< .001** | **<.05** |
| Trainees | 0.39 (0.32 - 0.45) | 0.85 (0.80 -0.90) | - | **< .001** | - |
