# Supplementary material for: Near-Infrared Autofluorescence for Parathyroid Detection During Endocrine Neck Surgery: A Randomized Clinical Trial
Source: JAMA Surg. 2025 Jul 16;160(9):936–44. doi: 10.1001/jamasurg.2025.2233 (PMC12268529; doi:10.1001/jamasurg.2025.2233)
Supplement: Supplement 2. — Data sharing statement [file jamasurg-e252233-s002.pdf]

Data Sharing Statement

Cousart. Near-Infrared Autofluorescence for Parathyroid Detection During Endocrine Neck Surgery. *JAMA Surg*. Published July 16, 2025. doi:10.1001/jamasurg.2025.2233

Data
Additional Information: NCT05579782, NCT05022667, NCT05022641, NCT04281875, NCT04299425, NCT05152927
Data available: Yes
Data types: Deidentified participant data
How to access data: The proposed use of this data should be directed to the corresponding author, Dr. Anita Mahadevan-Jansen (anita.mahadevan-jansen@vanderbilt.edu).
When available: With publication

Supporting Documents
Document types: Statistical/analytic code
How to access documents: The proposed use of this data should be directed to the corresponding author, Dr. Anita Mahadevan-Jansen (anita.mahadevan-jansen@vanderbilt.edu).
When available: With publication

Additional Information
Who can access the data: Researchers with a methodologically sound proposal
Types of analyses: To achieve aims in the approved proposal.
Mechanisms of data availability: The proposed use of this data should be directed to the corresponding author, Dr. Anita Mahadevan-Jansen (anita.mahadevan-jansen@vanderbilt.edu).
